# An analysis of volumes, prices and pricing trends of the pediatric antiretroviral market in developing countries from 2004 to 2012

**DOI:** 10.1186/s12887-016-0578-x

**Published:** 2016-03-15

**Authors:** Janice Soo Fern Lee, Luis Sagaon Teyssier, Boniface Dongmo Nguimfack, Intira Jeannie Collins, Marc Lallemant, Joseph Perriens, Jean-Paul Moatti

**Affiliations:** 1 Drugs for Neglected Diseases initiative (DNDi), 15 Chemin Louis Dunant, 1202 Geneva, Switzerland; 2 INSERM, UMR912 “Economics and Social Sciences Applied to Health & Analysis of Medical Information” (SESSTIM), 13006 Marseille, France; 3 Aix Marseille University, UMR_S912, IRD, 13006 Marseille, France; 4 ORS PACA, Southeastern Health Regional Observatory, 13006 Marseille, France; 5 HIV Department, World Health Organization, Geneva, Switzerland; 6 Medical Research Council Clinical Trials Unit, Institute of Clinical Trials and Methodology, University College London, London, UK

**Keywords:** Pediatrics antiretroviral market, Pediatric antiretroviral prices, Global Price Reporting Mechanism, Price trends, Pediatric antiretroviral procurement

## Abstract

**Background:**

The pediatric antiretroviral (ARV) market is poorly described in the literature, resulting in gaps in understanding treatment access. We analyzed the pediatric ARV market from 2004 to 2012 and assessed pricing trends and associated factors.

**Methods:**

Data on donor funded procurements of pediatric ARV formulations reported to the Global Price Reporting Mechanism database from 2004 to 2012 were analyzed.

Outcomes of interest were the volume and mean price per patient-year ARV formulation based on WHO ARV dosing recommendations for a 10 kg child. Factors associated with the price of formulations were assessed using linear regression; potential predictors included: country income classification, geographical region, market segment (originator versus generic ARVs), and number of manufacturers per formulation. All analyses were adjusted for type of formulations (single, dual or triple fixed-dose combinations (FDCs))

**Results:**

Data from 111 countries from 2004 to 2012 were included, with procurement of 33 formulations at a total value of USD 204 million. Use of dual and triple FDC formulations increased substantially over time, but with limited changes in price. Upon multivariate analysis, prices of originator formulations were found to be on average 72 % higher than generics (*p* < 0.001). A 10 % increase in procurement volume was associated with a 1 % decrease (*p* < 0.001) in both originator and generic prices. The entry of one additional manufacturer producing a formulation was associated with a decrease in prices of 2 % (*p* < 0.001) and 8 % (*p* < 0.001) for originator and generic formulations, respectively. The mean generic ARV price did not differ by country income level. Prices of originator ARVs were 48 % (*p* < 0.001) and 14 % (*p* < 0.001) higher in upper-middle income and lower-middle income countries compared to low income countries respectively, with the exception of South Africa, which had lower prices despite being an upper-middle income country.

**Conclusions:**

The donor funded pediatric ARV market as represented by the GPRM database is small, and lacks price competition. It is dominated by generic drugs due to the lower prices offered and the practicality of FDC formulations. This market requires continued donor support and the current initiatives to protect it are important to ensure market viability, especially if new formulations are to be introduced in the future.

## Background

In 2012, 3.4 million children were living with HIV/AIDS, 90 % of whom were in sub-Saharan Africa and only 647,000 were receiving antiretroviral (ARV) therapy [1]. For several years, the World Health Organization (WHO) has recommended early diagnosis and immediate treatment with ARVs for all children under two years of age irrespective of CD4 count, and since June 2013, for all children under five years of age [2], meaning that at the end of 2012, 2.6 million children who were eligible for treatment did not receive it.

Research and development for pediatric ARVs has been slow. Of the 26 ARVs approved by the United States Food and Drugs Administration (USFDA) and marketed, 7 have no pediatric indication, 8 have no pediatric formulation, and only 11 are approved for use in children below two years of age [3]. In the early years of combined ARV therapy, this lack of appropriate formulations meant that programs in resource limited settings had to resort to breaking adult fixed dose combination (FDC) tablets to treat children [4, 5].

In response to the need for pediatric FDCs, a WHO/ United Nations Children’s Fund (UNICEF) consultation in 2004 established a priority list of missing formulations and discussed ways to engage pharmaceutical companies to produce them [6]. Further consultations updated the list of ARVs to be developed, and identified key research areas to further facilitate FDC development [7, 8]. Other milestones include having these formulations listed on the WHO Prequalification Project’s Expression of Interest and subsequently on the Essential Medicines List, thus enabling developing countries to purchase quality assured generic ARVs, often a requirement from international donors.

Since 2006, UNITAID, an organization dedicated to providing funds to address market failures in the fight against HIV/AIDS, malaria and tuberculosis in developing countries, successfully incentivized generic companies to produce the “missing” ARV formulations [9]. By pooling procurement across 40 countries and committing to purchase ARVs, it created a market for pediatric FDCs and became the largest provider for developing countries (97–100 % of the pediatric market-share by 2008–2009) [10]. In 2010, much of the pediatric antiretroviral procurement responsibility was transitioned to other donors, in particular the Global Fund to Fight AIDS, Tuberculosis and Malaria (GFATM) [11].

In October 2011, the Joint United Nations Programme on HIV/AIDS (UNAIDS) and its partners launched the Global Plan Towards the Elimination of New HIV Infections Among Children by 2015 [12]. Although this initiative provided considerable momentum for the prevention of new infections, WHO forecasted that 1.9 million children will be living with HIV in 2020, with an estimated 1.6 million in need of antiretroviral treatment (ART) [13].

A first analysis of the pediatric ARV market was published in 2010 which focused on the availability and use of pediatric formulations between 2002 and 2009 [10]. The analysis gave an overview of pediatric formulations conforming to WHO recommendations and usage of formulations following WHO prequalification program or USFDA (tentative) approval. Little was reported on pricing trends across regions and formulations. Our analysis seeks to fill the knowledge gap since then, given that WHO guidelines have changed, new formulations have been introduced, and the factors associated with price trends of pediatric ARV formulations are largely unknown. We present our findings using the WHO’s Global Price Reporting Mechanism (GPRM) database which has been tracking international transactions of HIV, tuberculosis and malaria commodities purchased by national programmes in low- and middle-income countries through international procurement organizations since 2004. This database represents about 80 % of total donor-funded transactions worldwide [14].

## Methods

The GPRM database contains information about prices and volumes of each individual transaction, dosage form and strength of formulations, manufacturers, procurement agents, destination countries, international commercial terms (INCOTERMS), and procurement dates obtained from 11 procurement organizations on a quarterly basis. The analyses were based on GPRM data collected between 2004 and 2012. Prices are reported in current USD. To remove variability arising from the use of different INCOTERMS and to allow comparability, prices were expressed in Ex Works (price of goods at Seller’s premises, the Buyer bearing full costs and risks of moving the goods from there to destination) using a published statistical algorithm [15]. For each of the 21 ARV single formulations, 7 dual FDCs, and 5 triple FDCs , we calculated the quantity per year (QTY) and price per year (PTY) using WHO ARV dosing recommendations for a 10 kg child (2004–2005 dosing based on WHO 2002 guidelines, 2006–2009 dosing based on WHO 2006 guidelines, and 2010–2012 dosing based on WHO 2010 guidelines):

$$ \mathbf{Q}\mathbf{T}\mathbf{Y} = \left(\mathrm{number}\ \mathrm{of}\ \mathrm{units}\ \mathrm{purchased}\right)/\left[\left(\mathrm{units}\ \mathrm{used}\ \mathrm{in}\ \mathrm{daily}\ \mathrm{treatment}\right) \times (365)\right] $$

and

$$ \mathbf{P}\mathbf{T}\mathbf{Y} = \left(\mathrm{unit}\ \mathrm{price}\ \mathrm{US}\$\right) \times \left(\mathrm{unit}\mathrm{s}\ \mathrm{used}\ \mathrm{in}\ \mathrm{daily}\ \mathrm{treatment}\right) \times (365). $$

Countries were grouped into 7 geographic areas: East Asia and Pacific, Europe and Central Asia, Latin-America and the Caribbean, Middle East and North Africa, South Asia, sub-Saharan Africa excluding South Africa, and South Africa. South Africa was separated from sub-Saharan Africa in the analysis due to the large volume of drugs purchased by the country which could have confounded the outcomes for the sub-Saharan Africa region as a whole. Countries were also grouped by Gross National Income (GNI) per capita using World Bank classifications of low-income, lower-middle-income, and upper-middle income economies. GNI classifications were revised yearly. Formulations were classified into single ARV, double FDCs and triple FDCs.

### Descriptive analysis of volumes and prices of pediatric formulation procurement

We analyzed the evolution of volumes procured, by region and by country income levels for originator and generic products, for single ARVs, dual FDCs and triple FDCs; and the change in mean prices of single ARVs, dual and triple FDCs over time.

### Multivariate analysis of the factors associated with the price of pediatric antiretrovirals

We used a linear regression model to assess the factors associated with the price of formulations, with fixed-effects for calendar time and geographical regions. The outcome of formulation patient-year cost was the dependent variable. It was transformed into its natural logarithm in order to facilitate the interpretation of coefficients as percentages of variation. The potential factors associated with prices included in the model were: originator versus generic producers, country income class, geographical region, type of formulation (single ARV, dual FDC, triple FDC), number of suppliers and purchase volume.

## Results

The numbers of countries contributing data increased from 46 in 2004 to 111 in 2012. Over the observed time period, there were 33 formulations, 15 162 transactions, 2 447 252 QTY and a total purchasing value of USD 204 million.

**Fig. 1:**
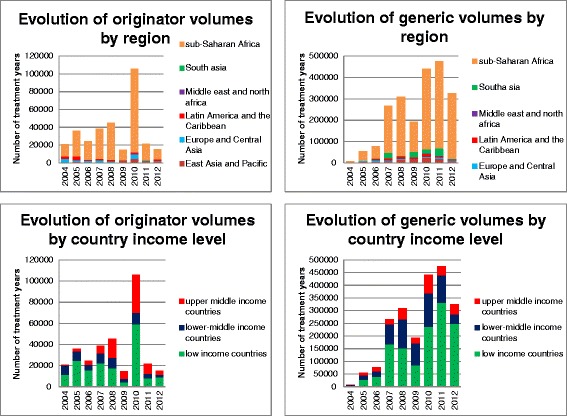
Evolution of treatment volumes by region and country income levels for originator and generic products

**Fig. 2:**
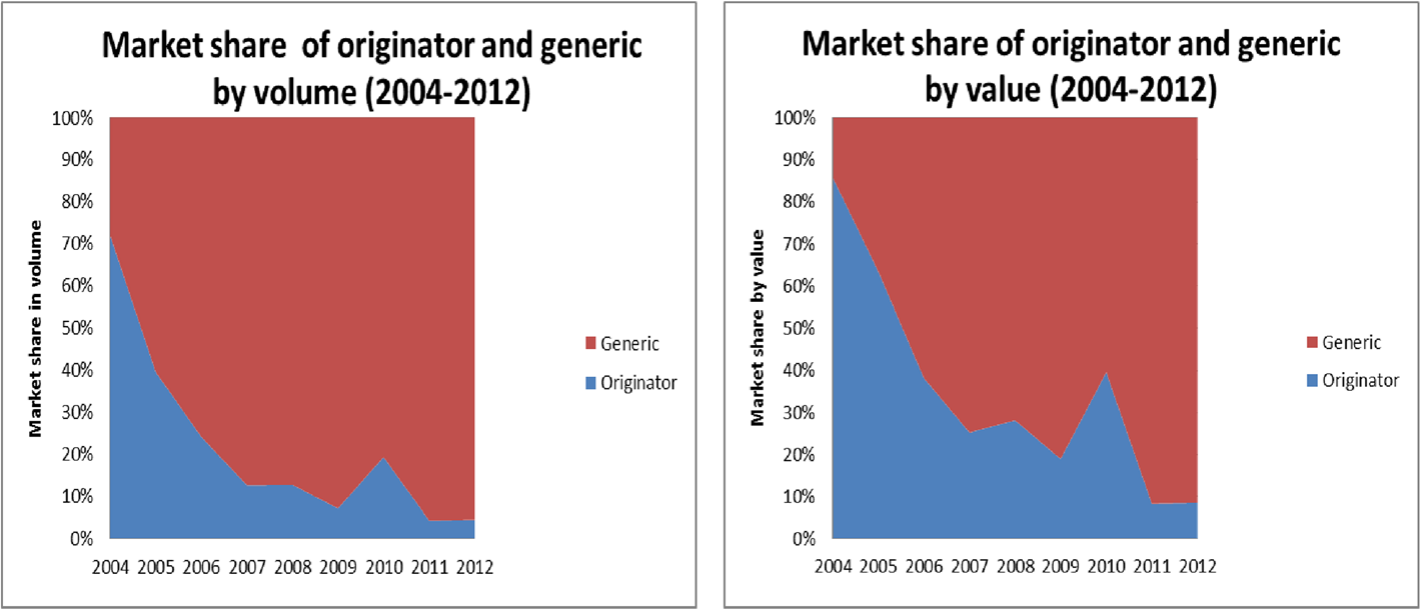
Market share of generic and originator ARVs by volume and price

**Fig. 3:**
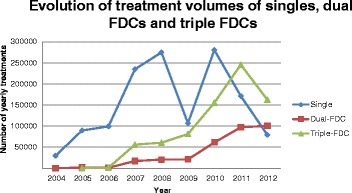
Evolution of treatment volumes of singles, dual FDCs and triple FDCs

From 2004 to 2012, sub-Saharan Africa represented 85 % of the total volume of pediatric ARVs purchased from both originator and generic manufacturers (Fig. 1). The market was originally dominated by originator companies with 72 % of the volume purchased in 2004 (Fig. 2). Since 2005, generic companies have taken over, accounting for 95 % of volume and 92 % of value in 2012. Use of dual and triple FDCs has increased markedly since 2009, with single ARV volumes decreasing from 2010 onwards. Triple FDCs recorded their highest purchase volume in 2012, followed by dual FDC and single ARVs (Fig. 3). It is worth noting that, with the exception of lopinavir/ritonavir (LPV/r), pediatric dual FDCs and triple FDCs were exclusively produced by generic companies, while single ARVs and LPV/r were produced by both.

**Fig. 4:**
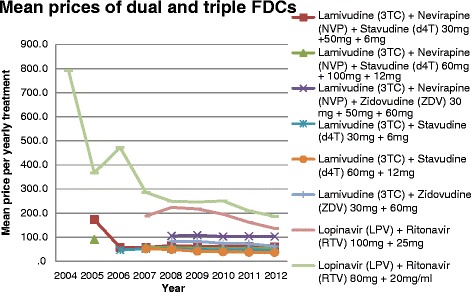
Evolution of mean prices of dual FDCs and triple FDCs

Generally, the prices of single ARVs have decreased since 2004. However, the prices of dual and triple FDCs have remained almost constant after their first year post-introduction (Fig. 4). By 2012 the transaction volume of zidovudine/lamivudine/nevirapine had increased 12-fold since its entry into the market in 2008.

Upon multivariate analysis, prices of originator formulations were on average 72 % higher than generics (*p* < 0.001) (Table 1). The prices of generic ARVs were 54 % lower in 2012 compared to 2004 (*p* < 0.001), however the majority of this price reduction had occurred by 2006, with limited change thereafter. Overall, originator prices were 52 % lower in 2012 than in 2004 (*p* < 0.001).

**Table 1:** Multivariate analysis of the factors associated with prices^d^ of pediatric antiretrovirals

	ALL (*n* = 15,162)		Originator (*n* = 5362)		Generic (*n* = 9800)	
	Estimate	95 % CI	Estimate	95 % CI	Estimate	95 % CI
*Years (Analysis performed in comparison to 2004)*						
2005	−0.06	[−0.14, 0.02]	0.00	[−0.10, 0.09]	−0.13	[−0.27, 0.00]
2006	−0.21^c^	[−0.29, −0.13]	−0.14^b^	[−0.24, −0.04]	−0.30^c^	[−0.44, −0.17]
2007	−0.33^c^	[−0.40, −0.26]	−0.1^c^	[−0.28, −0.10]	−0.46^c^	[−0.58, −0.34]
2008	−0.45^c^	[−0.52, −0.38]	−0.27^c^	[−0.36, −0.19]	−0.56^c^	[−0.68, −0.44]
2009	−0.49^c^	[−0.56, −0.41]	−0.33^c^	[−0.43, −0.23]	−0.60^c^	[−0.73, −0.47]
2010	−0.50^c^	[−0.57, −0.43]	−0.29^c^	[−0.38, −0.19]	−0.62^c^	[−0.75, −0.50]
2011	−0.45^c^	[−0.53, −0.38]	−0.45^c^	[−0.56, −0.35]	−0.50^c^	[−0.62, −0.38]
2012	−0.48^c^	[−0.56, −0.41]	−0.52^c^	[−0.63, −0.41]	−0.54^c^	[−0.66, −0.41]
*Geographical regions (Analysis performed in comparison to sub-Saharan Africa excluding South Africa)*						
East Asia and Pacific	−0.03	[−0.07, 0.02]	0.26^c^	[0.17, 0.35]	−0.11^c^	[−0.16, −0.06]
Europe and Central Asia	0.28^c^	[0.22, 0.33]	0.66^c^	[0.56, 0.76]	0.08^a^	[0.02, 0.14]
Latin America and the Caribbean	0.07^b^	[0.02, 0.11]	0.31^c^	[0.23, 0.38]	0.00	[−0.05, 0.05]
Middle East and North Africa	0.08	[0.00, 0.17]	0.29^c^	[0.14, 0.44]	−0.05	[−0.14, 0.05]
South Asia	−0.13^c^	[−0.20, −0.06]	0.13	[−0.18, 0.44]	−0.13^c^	[−0.21, −0.06]
South Africa	−0.23^c^	[−0.29, −0.18]	−0.71^c^	[−0.81, −0.61]	0.24^c^	[0.17, 0.31]
*Income group (Analysis done in comparison to low income countries)*						
Lower-middle income countries	0.04^b^	[0.02, 0.07]	0.14^c^	[0.09, 0.20]	0.02	[−0.01, 0.05]
Upper-middle income countries	0.17^c^	[0.12, 0.22]	0.48^c^	[0.39, 0.58]	0.02	[−0.04, 0.07]
*Formulation type (Analysis done in comparison with single ARVs)*						
Double	0.01	[−0.04, 0.05]	0.10^c^	[0.05, 0.16]	−0.35^c^	[−0.42, −0.29]
Triple	−0.08^b^	[−0.15, −0.02]			−0.48^c^	[−0.56, −0.40]
*Effects of competition and volume*						
1 additional manufacturer	−0.04^c^	[−0.04, −0.03]	−0.02^c^	[−0.03, −0.01]	−0.08^c^	[−0.09, −0.07]
Log(quantity purchased)	−0.10^c^	[−0.10, −0.09]	−0.11^c^	[−0.12, −0.10]	−0.10^c^	[−0.10, −0.09]
*Market segment (analysis done in comparison to generic)*						
Originator	0.72^c^	[0.70, 0.75]				
Adjusted R^2^	0.35		0.20		0.16	
Sum of squared residuals	7413		2497		4404	

^a^significance level 0.05

^b^significance level 0.01

^c^significance level 0.001

^d^Current USD

There is a modest association between volume and ARVs prices, with a 10 % increase in volume associated with a 1 % decrease (*p* < 0.001) for both originator and generic prices. The number of manufacturers for a given formulation was limited, with 1–2 manufacturers for dual/triple FDCs, and 3–4 for single ARV formulations. The number of manufacturers was also modestly associated with price changes, with additional manufacturers associated with a decrease of 2 % (*p* < 0.001) and 8 % (*p* < 0.001) in originator and generic ARV prices, respectively.

Investigating prices by geographical region, we found that sub-Saharan Africa (excluding South Africa) was paying the lowest price for originator ARVs. However, the price of originator formulations in South Africa was on average 71 % (*p* < 0.001) lower than in sub-Saharan Africa, essentially because of high volumes and potential price negotiations which could have taken place for formulations such as abacavir solution, lopinavir/ritonavir pediatric tablets and nevirapine suspension. Generic drugs formed 70 % of the total purchase volume in South Africa and their price was 24 % (*p* < 0.001) higher than the rest of sub-Saharan Africa. East Asia and Pacific and South Asia were paying 10 % (*p* < 0.001) and 13 % (*p* < 0.001) less than sub-Saharan Africa respectively.

Compared to low income countries, originator ARVs prices were 14 % (*p* < 0.001) and 48 % (*p* < 0.001) higher in lower-middle and upper-middle income countries respectively. Generic ARV prices within country classifications did not differ significantly.

## Discussion

Our multivariate analysis shows that originator prices are on average 72 % higher than generic prices, despite the marked decrease of 52 % in overall originator prices in 2012 compared to 2004 (*p* < 0.001.) It is therefore not surprising that this donor-dominated market was rapidly overtaken by generic products. In 2012, 95 % of pediatric ARVs were purchased from generic companies. Price was not the only factor influencing this change; the availability of child-friendly FDCs also played an important role. The prices of pediatric FDCs have remained stagnant despite the fact that volumes of triple and dual FDCs outstripped that of single ARV formulations in 2011. This may be explained by the fact that many organisations have advocated for pediatric FDCs. Even before the development of paediatric FDCs, Médecins sans Frontières reported good outcomes for children using adult FDC in resource limited settings and advocated for child friendly FDCs [5]. WHO and UNICEF further promoted pediatric FDCs through the development of treatment guidelines, priority lists of missing formulations and engaging manufacturers to stimulate product development. A final push was given by UNITAID, an organization financed by a solidarity levy on airline tickets. It successfully created a market for pediatric FDCs in 2006 with the announcement of a price deal of 16 cents a day per child for stavudine/lamivudine/nevirapine [16]. This price positioning was obtained through the advocacy efforts of large institutions and UNITAID’s commitment to purchase commodities.

With the exception of LPV/r, dual and triple pediatric FDCs are exclusively produced by generic manufacturers. They are produced in India where patents for medicines were not granted before 2005 [17]. Developing countries have access to these formulations because according to the Trade-Related Aspects of Intellectual Property Rights (TRIPS) Agreement, least developed countries do not have to enforce intellectual property rights until 2016 [18]. In the United States of America (USA), these pediatric FDCs were approved by the USFDA under a special program associated with the President’s Emergency Plan (PEPFAR); products with IP protection in the USA may be reviewed and receive “tentative approval” allowing them to be purchased under PEPFAR programs for use in developing countries, but with no marketing rights in the USA. While pediatric FDCs are now the cornerstone of treatment for children in developing countries, they are not available in developed countries where intellectual property (IP) barriers do not allow their commercialization.

Various terms have been used for the pricing strategy that originator pharmaceutical companies adopt in setting prices for countries with different income levels, such as “tiered pricing”, “differential pricing”, “market separation” and “price discrimination” [19–21]. This approach is reflected in the pricing trends of our analysis and may explain why low income countries are paying the lowest originator price, followed by lower-middle income and upper-middle income countries. Although the eligibility criteria for tiered pricing and the different categories of pricing vary across originator companies, 6 out of 7 originator companies include sub-Saharan African countries in their lowest tiered pricing category for ARVs [22, 23]. This explained why, with the exception of low income countries, sub-Saharan African countries also paid the lowest price of all geographical regions for originator ARVs. While the originator’s tiered pricing strategy generally matches prices with the country’s purchasing power, South Africa is an exception. We excluded South Africa from the sub-Saharan African countries in the analysis because it represents a substantial volume of purchase, South African tender favors the selection of local manufacturers and has a committee that specifically regulates pharmaceutical prices [24]. This upper-middle income country pays 71 % less for its originator drugs than the rest of sub-Saharan Africa.

For generic pediatric ARVs, sub-Saharan Africa (South Africa excluded) has not paid the lowest prices. East Asia and Pacific and South Asia were paying 11–13 % less for generic ARVs. The prices of generic ARVs across the 3 economic income groups were not significantly different. Generic pediatric ARV pricing does not appear not to be linked to country income levels or geographical region, suggesting a different pricing strategy to that of the originator companies.

To our knowledge, this is the first time that a thorough analysis of pricing trends of pediatric ARVs from 2004 to 2012 has been presented. While this database captures mostly donor related pediatric ARV transactions, it reflects almost 80 % of donor transactions worldwide. It is a good representation of the pediatric ARV market since 90 % of the children living with HIV are from sub-Saharan African countries where provision of ARVs is largely donor-funded. This analysis has several limitations that should be noted. It could not take into account ARVs for older children who can use adult formulations. In addition, it could not separate ARVs used for treatment and those used for prevention of mother-to-child transmission (PMTCT). However, this is likely to have a negligible effect since the use of paediatric ARVs for PMTCT is limited to two single ARVs, namely AZT or NVP liquid formulations [25–27]. It should be noted that this database represents procurement data and not actual consumption data, and that the quantity and prices calculated per formulation do not represent quantity and prices of actual treatment regimens. The use of Ex-Works prices in this analysis does not take into account other costs such as transportation, insurance, import duties and taxes. We have also noted differences in characteristics at a national level, such as domestic manufacturing capacity for some countries, but it would be difficult to incorporate these into a global level analysis as conducted here.

Another analysis of the GPRM database by Perriens et al. concluded that a great majority of pediatric ARV formulations are being sold at prices that are profitable when the prices were analysed with respect to active pharmaceutical ingredient (API) cost, provided that the cost of development can be recovered from sufficient sales volume [28]. Children represent only 6 % of the total number of people receiving ART in the 2012 WHO survey [29] making the pediatric ARV market a small and fragile market. The number of HIV infected children is dwindling due to the success of prevention programs, as evidenced by the number of children newly infected with HIV dropping from 520,000 in 2000 to 240,000 in 2013 [30]. With a general lack of competition as shown by the stagnation of prices in pediatric FDCs despite relatively high volume of procurement, the pediatric market contrasts with the adult market where prices have decreased drastically over time; the median price per treatment per year paid for adult first line treatment regimens in low and middle income countries decreased 5 fold between 2003 and 2012 [23, 28]. The pediatric market will become even smaller and more fragile if the scale-up of treatment for children does not happen rapidly. WHO recently recommended the development of 11 new pediatric formulations, at the risk of a lack of interest in their development by generic manufacturers who need to recoup research and development costs from the limited profit margins available in this small market [2]. Therefore there is an urgent need to prioritize and rationalize new formulation development with planned phasing out of redundant formulations.

Many initiatives are taking place at the global level to protect this market. In May 2011, a special pediatric working group from the Inter Agency Task Team on Prevention and Treatment of HIV Infection in Pregnant Women, Mothers and their Children produced a list of optimized pediatric ARV formulations to guide donors, ministries of health and procurement agencies to prioritize purchase of pediatric formulations [31]. In parallel, UNITAID, Global Fund, PEPFAR, UNICEF and other stakeholders have set up a Pediatric ARV Procurement Working Group to align procurement, promote product optimization, secure financing, engage with manufacturers and provide in-country support.

## Conclusions

The donor funded pediatric ARV market as represented by the GPRM database is small, and lacks price competition. It is dominated by generic drugs due to the lower prices offered and the practicality of FDC formulations. This market requires continued donor support and the current initiatives to protect it are important to ensure market viability, especially if new formulations are to be introduced in the future.

### Availability of supporting data

Global Price Reporting Mechanism database is accessible at http://apps.who.int/hiv/amds/price/hdd/.

## Abbreviations

ART: antiretroviral treatment
ARV: antiretroviral
FDC: fixed dose combination
GFATM: Global Fund to Fight AIDS, Tuberculosis and Malaria
GPRM: Global Price Reporting Mechanism
INCOTERMS: international commercial terms
IP: intellectual property
LPV/r: lopinavir/ritonavir
PEPFAR: President’s Emergency Plan
PTY: price per year
QTY: quantity per year
UNICEF: United Nations Children’s Fund
USFDA: United States Food and Drugs Administration
WHO: World Health Organization
